# A 36-week multicenter, randomized, double-blind, placebo-controlled, parallel-group, phase 3 clinical trial of sodium oligomannate for mild-to-moderate Alzheimer’s dementia

**DOI:** 10.1186/s13195-021-00795-7

**Published:** 2021-03-17

**Authors:** Shifu Xiao, Piu Chan, Tao Wang, Zhen Hong, Shuzhen Wang, Weihong Kuang, Jincai He, Xiaoping Pan, Yuying Zhou, Yong Ji, Luning Wang, Yan Cheng, Ying Peng, Qinyong Ye, Xiaoping Wang, Yuncheng Wu, Qiumin Qu, Shengdi Chen, Shuhua Li, Wei Chen, Jun Xu, Dantao Peng, Zhongxin Zhao, Yansheng Li, Junjian Zhang, Yifeng Du, Weixian Chen, Dongsheng Fan, Yong Yan, Xiaowei Liu, Wei Zhang, Benyan Luo, Wenyuan Wu, Lu Shen, Chunfeng Liu, Peixian Mao, Qiumei Wang, Qianhua Zhao, Qihao Guo, Yongtao Zhou, Yi Li, Lijun Jiang, Wenwei Ren, Yingjun Ouyang, Yan Wang, Shuai Liu, Jianjun Jia, Nan Zhang, Zhonglin Liu, Raoli He, Tingyi Feng, Wenhui Lu, Huidong Tang, Ping Gao, Yingchun Zhang, Lanlan Chen, Lei Wang, You Yin, Qun Xu, Jinsong Xiao, Lin Cong, Xi Cheng, Hui Zhang, Dan Gao, Minghua Xia, Tenghong Lian, Guoping Peng, Xu Zhang, Bin Jiao, Hua Hu, Xueyan Chen, Yihui Guan, Ruixue Cui, Qiu Huang, Xianliang Xin, Hongjian Chen, Yu Ding, Jing Zhang, Teng Feng, Marc Cantillon, Kewei Chen, Jeffrey L. Cummings, Jian Ding, Meiyu Geng, Zhenxin Zhang

**Affiliations:** 1 Department of Geriatric Psychiatry, Shanghai Mental Health Center, Shanghai Jiaotong University School of Medicine, Shanghai, China; 2 Alzheimer’s Disease and Related Disorders Center, Shanghai Jiaotong University, 600 South Wan Ping Road, Shanghai, 200030 China; 3 Xuanwu Hospital Capital Medical University, Beijing, China; 4 Huashan Hospital, Fudan University, Shanghai, China; 5 Qilu Hospital of Shandong University, Ji’nan, China; 6 West China Hospital of Sichuan University, Chengdu, China; 7 The First Affiliated Hospital of Wenzhou Medical University, Wenzhou, China; 8 Guangzhou First People’s Hospital, School of Medicine, South China University of Technology, Guangzhou, China; 9 Tianjin Huanhu Hospital, Huanhu Hospital Affiliated to Nankai University, Tianjin, China; 10 Department of Geriatric Neurology of PLA General Hospital, Beijing, China; 11 Department of Neurology, Tianjin Medical University General Hospital, Tianjin, China; 12 Sun Yat-Sen Memorial Hospital, Sun Yat-Sen University, Guangzhou, China; 13 Fujian Medical University Union Hospital, Fuzhou, China; 14 Department of Neurology, Shanghai General Hospital, Shanghai Jiao Tong University School of Medicine, Shanghai, China; 15 Department of Neurology, The First Affiliated Hospital of Xi’an Jiaotong University, Xi’an, China; 16 Department of Neurology, Ruijin Hospital, Shanghai Jiaotong University School of Medicine, Shanghai, China; 17 Beijing Hospital, Beijing, China; 18 Department of Psychiatry, Sir Run Run Shaw Hospital, Zhejiang University School of Medicine and Key Laboratory of Medical Neurobiology of Zhejiang Province, Hangzhou, China; 19 Northern Jiangsu People’s Hospital, Yangzhou, China; 20 Department of Neurology, China-Japan Friendship Hospital, Beijing, China; 21 Shanghai Changzheng Hospital, Shanghai, China; 22 Renji Hospital, Shanghai Jiaotong University School of Medicine, Shanghai, China; 23 Department of Neurology, Zhongnan Hospital of Wuhan University, Wuhan, China; 24 Shandong Provinical Hospital affiliated to Shandong University, Ji’nan, China; 25 Jiangsu Province People’s Hospital, Nanjing, China; 26 Peking University Third Hospital, Beijing, China; 27 The First Affiliated Hospital of Chongqing Medical University, Chongqing, China; 28 Department of Geriatric psychiatry, Wuxi Mental Health Center, Wuxi, China; 29 Department of Neurology, Beijing Tiantan Hospital, Capital Medical University, Beijing, China; 30 The First Affiliated Hospital, Zhejiang University School of Medicine, Hangzhou, China; 31 Tongji Hospital of Tongji University, Shanghai, China; 32 Xiangya Hospital Central South University, Changsha, China; 33 The Second Affiliated Hospital of Soochow University, Suzhou, China; 34 Beijing An Ding Hospital, Capital Medical University, Beijing, China; 35 Peking Union Medical College Hospital, No. 1 Shuaifuyuan, Beijing, 100730 China; 36 Med-X Research Institution, Shanghai Jiao Tong University, Shanghai, China; 37 Shanghai Green Valley Pharmaceutical Co. Ltd., No. 421, Niudun Road, Shanghai, China; 38 Banner Alzheimer’s Institute, Phoenix, AZ USA; 39 Chamberrs-Grundy Center for Transformative Neuroscience, Department of Brain Health, School of Integrated Health Sciences, University of Nevada, Las Vegas, USA; 40 State Key Laboratory of Drug Research, Shanghai Institute of Materia Medica, Chinese Academy of Sciences, 555 Zu chong zhi Road, Nevada, China

**Keywords:** Sodium oligomannate, Efficacy, Safety, Alzheimer’s disease, Clinical trial

## Abstract

**Background:**

New therapies are urgently needed for Alzheimer’s disease (AD). Sodium oligomannate (GV-971) is a marine-derived oligosaccharide with a novel proposed mechanism of action. The first phase 3 clinical trial of GV-971 has been completed in China.

**Methods:**

We conducted a phase 3, double-blind, placebo-controlled trial in participants with mild-to-moderate AD to assess GV-971 efficacy and safety. Participants were randomized to placebo or GV-971 (900 mg) for 36 weeks. The primary outcome was the drug-placebo difference in change from baseline on the 12-item cognitive subscale of the Alzheimer’s Disease Assessment Scale (ADAS-cog12). Secondary endpoints were drug-placebo differences on the Clinician’s Interview-Based Impression of Change with caregiver input (CIBIC+), Alzheimer’s Disease Cooperative Study-Activities of Daily Living (ADCS-ADL) scale, and Neuropsychiatric Inventory (NPI). Safety and tolerability were monitored.

**Results:**

A total of 818 participants were randomized: 408 to GV-971 and 410 to placebo. A significant drug-placebo difference on the ADAS-Cog12 favoring GV-971 was present at each measurement time point, measurable at the week 4 visit and continuing throughout the trial. The difference between the groups in change from baseline was − 2.15 points (95% confidence interval, − 3.07 to − 1.23; *p* < 0.0001; effect size 0.531) after 36 weeks of treatment. Treatment-emergent adverse event incidence was comparable between active treatment and placebo (73.9%, 75.4%). Two deaths determined to be unrelated to drug effects occurred in the GV-971 group.

**Conclusions:**

GV-971 demonstrated significant efficacy in improving cognition with sustained improvement across all observation periods of a 36-week trial. GV-971 was safe and well-tolerated.

**Trial registration:**

ClinicalTrials.gov, NCT02293915. Registered on November 19, 2014

**Supplementary Information:**

The online version contains supplementary material available at 10.1186/s13195-021-00795-7.

## Background

Alzheimer’s disease (AD) is the most common type of dementia in older people. According to the *World Alzheimer Report 2018*, approximately 50 million people worldwide are currently suffering from dementia, and two thirds of them have AD. China has more than 6 million AD patients, and this number is expected to exceed 10 million by 2050 [1, 2]. AD has become a major cause of disability and death among older individuals. AD has a devastating social and economic impact on patients, their families, their caregivers, medical systems, and society. Several hundred treatments for AD have been tested in clinical trials, but only four [3–6] have been authorized and widely used for AD treatment. Although repeated failures of phase 3 clinical trials in the last decade have been reported [7, 8], much effort continues to be invested in developing treatments for this disease [9].

Sodium oligomannate (GV-971) is a marine-derived oligosaccharide and a mixture of linear, acidic oligosaccharides with a degree of polymerization ranging from dimers to decamers [10]. After oral administration, most of the ingested GV-971 is retained in the gut. It is proposed that it can reconstitute the gut microbiota, reduce bacterial metabolite-driven peripheral infiltration of immune cells into the brain, and inhibit neuroinflammation in the brain as observed in animal models [11]. Some GV-971 can penetrate into the brain and directly inhibit Aβ fibril formation and destabilizes the preformed fibrils into non-toxic monomers. It reduces Aβ deposition in the brain of Aβ-transgenic mice [12–15]. The reduction in both Aβ deposition and neuroinflammation may synergistically contribute to the improvement of cognitive function observed in multiple non-clinical models. Although the above-proposed mechanism of action requires validation in humans, the non-clinical mechanistic studies have demonstrated that the properties of GV-971 make it a candidate anti-AD therapy. Phase 1 and phase 2 studies demonstrated that GV-971 is safe and well-tolerated, and 450 mg twice-a-day (b.i.d.) was selected as phase 3 dosage [16].

Based on the results and experience from the phase 2 trial, we designed and completed the phase 3 trial. The phase 3 trial reported here was undertaken to further evaluate the efficacy and safety of GV-971 in participants with mild-to-moderate AD.

## Methods

### Study design

This phase 3 study was a 36-week multicenter, randomized, double-blind, placebo-controlled parallel-group clinical trial. Participants with mild-to-moderate AD were assigned randomly (1:1 ratio), to receive GV-971 (450 mg, b.i.d.) or placebo.

The trial protocol was approved by the Ethics Review Board of Shanghai Mental Health Center (Shanghai, China) and is registered on https://clinicaltrials.gov/NCT02293915. The protocol was also approved by the Institutional Review Boards of all participating sites. The Clinical New Drug Research and Development Team of the Department of Psychogeriatrics of Shanghai Mental Health Center and the sponsor designed the study in consultation with academic advisors. All participants or their representatives provided written informed consent before participation in the trial.

Data were gathered by the study investigators through the Merge system (www.merge.com/eClinical), analyzed by IQVIA (Durham, NC, USA) after the data were locked, and interpreted by the academic main investigators in collaboration with the sponsor. The academic authors attest to the accuracy and integrity of the data and the fidelity of this report to the study protocols, which are available with the full protocol (protocol number: 971-III; version 7.1) and statistical analysis plan (supplementary appendix).

There were 8 protocol amendments and 4 protocol versions in the course of the trial. A planned 24-week interim analysis was deleted, and the study continued to its primary outcome at 36 weeks. The other amendments included deletion of the age adjustments on the interpretation of the Fazekas scale of allowable white matter pathology on magnetic resonance imaging (MRI) and adjustment in how routine laboratory studies were interpreted. The subject whose Mini-Mental State Examination (MMSE) [17] fluctuation was more than 2 score from screening visit to randomize visit should be double-checked according to the inclusion/exclusion criteria in order to accurately enroll the subjects participant. Allowable heart rates were broadened (to > 55 beats per minute), and apolipoprotein E (APOE) genotyping was made mandatory. The intended sample size of 788 was somewhat over-recruited resulting in a final sample size of 818 randomized subjects.

### Participants

Participants were aged 50–85 years of age and met the diagnostic criteria for probable AD according to the National Institute of Neurological and Communicative Disorders and Stroke and the Alzheimer’s Disease and Related Disorders Association [18]. Participants had mild-to-moderate AD, with a MMSE score from 11 to 22 inclusive for participants with only a primary school education and from 11 to 26 inclusive for those with an education beyond primary school, in line with representative China national sample of age, gender, education reference norms [17, 19]. A total Hachinski Ischemia Scale [20] score of ≤4 was required, and the Hamilton Hamilton Depression Scale 17 [21] score had to be ≤10.

We strengthened brain MRI assessment in the phase 3 trial. During the screening, brain magnetic resonance imaging (MRI) with oblique coronal hippocampus images was undertaken; a medial temporal lobe atrophy visual rating scale score ≥ 2 was required [22]. MRI data were sent to an imaging advisory team via email in digital imaging and communications in medicine format along with a brief medical history. A Fazekas scale [23] for white matter lesions of grade ≥ 3 (moderate-to-severe) or > 2 lacunar infarction lesions of diameter 1.0–2.0 cm or infarction lesions in vital brain areas (thalamus, hippocampus, or entorhinal cortex) excluded participation. The brain MRI results of all participants were presented for diagnostic review by the imaging advisory team to minimize diagnostic error. The investigators reviewed the laboratory examination results and comments from the imaging advisory team on the brain MRI examination to ascertain if participants met the enrollment criteria.

Female participants were postmenopausal (last menopause at least 24 weeks prior to study entry), surgically sterilized, or of childbearing age who agreed to use contraceptive measures during the trial. Women of childbearing age and women < 24 weeks from the start of menopause underwent a urine pregnancy test during screening, and the result was required to be negative for study entry.

Participants were required to have completed education of at least primary school level or higher and be able to complete the protocol-specified cognitive tests. Progressive impairment of memory must have been present for ≥12 months. Care partners for the participants had frequent contact (≥4 days every week for ≥2 h on each of these days). Caregivers had to be willing to provide critical trial data on caregiver-based scales: CIBIC+ [24, 25], Alzheimer’s Disease Cooperative Study-Activities of Daily Living (ADCS-ADL) scale [26], and Neuropsychiatric Inventory (NPI) [27, 28]. Before implementation of a protocol-related procedure or examination, participants and caregivers provided written informed consent. If participants could not sign due to limited cognition, their legal guardians signed on their behalf.

Participants were excluded if they had taken part in another clinical trial < 30 days before the initiation of this trial or had dementia due to non-AD causes. Participants had a normal neurologic examination. They were excluded if they had abnormal laboratory values. They were also excluded if they had unstable or severe cardiac, pulmonary, hepatic, renal, or hematopoietic disease (including unstable angina, uncontrolled asthma, active gastric bleeding, or cancer); a resting heart rate < 55 bpm after 10 min of rest; a visual/hearing disorder that prevented completion of neuropsychologic tests and scale evaluations; or a history of alcohol abuse, drug abuse, or severe psychopathology (including major depression). Participants could not be on cholinesterase inhibitors or memantine while enrolled in this trial and were required to be off these agents for ≥4 weeks before randomization. The investigators excluded participants who they thought could not complete this trial, who participated in the phase 2 GV-971 trial, who were on AD therapies that could not be stopped, or were relatives of staff of IQVIA or Shanghai Green Valley Pharmaceuticals.

This clinical trial was conducted at 34 participating sites in the psychiatry, neurology, and geriatric departments of hospitals in several regions of China. [^18^F]-FDG-PET was carried out at two sites (Beijing and Shanghai) where appropriate technology and expertise were routinely available.

### Interventions

One capsule provides 150 mg of GV-971. The placebo is identical to the GV-971 capsule with regard to taste, odor, appearance, and design and is acceptable based on the quality standard inspection approved by the National Medical Products Administration (NMPA). GV-971 capsules are manufactured according to the Good Manufacturing Practice (GMP).

Two groups were intervened by GV-971 and placebo. The study comprised a 2-week screening period, 4-week run-in period, and 36-week treatment period. During the run-in period, each participant took three placebo capsules b.i.d. During the 36-week treatment period, each participant took three GV-971 capsules (150 mg/capsule) or three placebo capsules b.i.d.

Drug distribution was undertaken according to the drug randomization number generated by an interactive web response system (IWRS). After participants were determined to be eligible for trial participation, investigators logged into the IWRS system to obtain a randomization number and drug kit number for each participant. During the 36-week treatment period, participants were randomized (1:1 ratio) to receive GV-971 (450 mg, b.i.d.) or the matching placebo. During each on-site visit (Figure S3), investigators recorded the distribution and return of trial drugs. Participants, caregivers, and trial staff remained blinded to treatment assignment throughout the trial.

### Outcomes

The primary efficacy endpoint was the drug–placebo difference in the change from baseline on the ADAS-Cog12 (score range, 0–75, with higher scores indicating greater cognitive impairment) at week 36 [29]. Secondary endpoints were intergroup differences including CIBIC+ [24, 25] and changes from baseline in the ADCS-ADL scale (score range, 0–78, with lower scores indicating worse functioning) [26] and NPI (score range, 0–144, with lower scores indicating fewer behavioral disturbances) [27, 28]. Intergroup differences in change from baseline in a global index defined for relative cerebral metabolic rate for glucose (CMRglu) on [^18^F]-FDG PET [30] after 36 weeks of double-blind treatment was measured in a subgroup of participants. There was no change in the choice of and prioritization of outcome measures in the course of the trial.

From the time the subject signs the informed consent form, all adverse events (AEs) that occurred were captured on case report forms and included in the summaries. AE evaluation comprised classification of the organ system, grade, relationship to drug exposure, action taken to the treatment (if any), and the outcome.

Ten on-site visits and four telephone interviews were conducted with each participant. The on-site visits occurred at weeks − 6 and − 4, day 0, and weeks 4, 8, 12, 16, 24, 36, and 40. Telephone interviews were undertaken at weeks 2, 20, 28, and 32.

### Sample size

The sample size was calculated with nQuery Advisor 7.0. According to the results of the GV-971 phase 2 trial and other anti-AD clinical trials, a minimum ADAS-Cog drug-placebo difference of 1.4 was assumed. Completion of the trial by 315 participants in each arm would provide 80% power to detect a standardized effect size (difference/SD) of ≥0.233 between the GV-971 and placebo groups with a two-sided alpha level of 0.05. Therefore, we planned to enroll 788 participants, allowing for an anticipated 20% dropout rate. In total, 818 participants were randomized.

### Data statistics and study parameters

The primary endpoint (change from baseline of the ADAS-Cog12 score at week 36 in the treatment group compared to the placebo group) was analyzed using analysis of covariance (ANCOVA) with the treatment group, education level, pooled center, and age group as fixed effects and the baseline MMSE score and baseline ADAS-Cog12 score as covariates. Control-based pattern imputation was used to supply the missing data in the primary analysis. In general, this method assumed that, after trial withdrawal, participants from the treatment arm would exhibit the same future evolution of the disease as participants receiving the control treatment. Participants who discontinued the trial from the control arm were assumed to evolve in the same way as control participants who remained in the trial. In addition, a mixed-model repeated-measures model (MMRM) was used for sensitivity analyses. The primary efficacy analysis was based on the full analysis set (FAS), which included all randomly assigned participants who received at least one dose of the double-blind study drug and had a baseline value and at least one post-baseline efficacy assessment.

For secondary endpoints, CIBIC+ was analyzed by a stratified Cochran-Mantel-Haenszel test, including the stratification factors of MMSE at baseline, education level, and age group. ANCOVA was used to assess the drug-placebo difference in the change from baseline on ADCS-ADL and NPI scores.

Safety analyses were based on the safety analysis set, which included all randomly assigned participants who received at least one dose of a study agent. Safety analyses were based on a summary of AEs, laboratory assessments, electrocardiograms, vital signs, and physical examinations.

Participants in the cerebral [^18^F]-FDG-PET sub-cohort underwent PET at baseline and week 36. We used the statistical region of interest (sROI) approach to compute the relative global CMRglu global index [30]. sROI was introduced based on cross-validation and ADNI data and was used in a recent clinical trial [31]. The sROI-defined global CMRglu index is actually the standard uptake value ratio between a set of regions affected by AD and a set of regions spared by AD in terms of glucose uptake decline over time [30].

SAS v9.4 (SAS Institute, Cary, NC, USA) was used for statistical analyses. *p* < 0.05 (two-sided) indicated significance.

## Results

### Participant flow

**Fig. 1:**
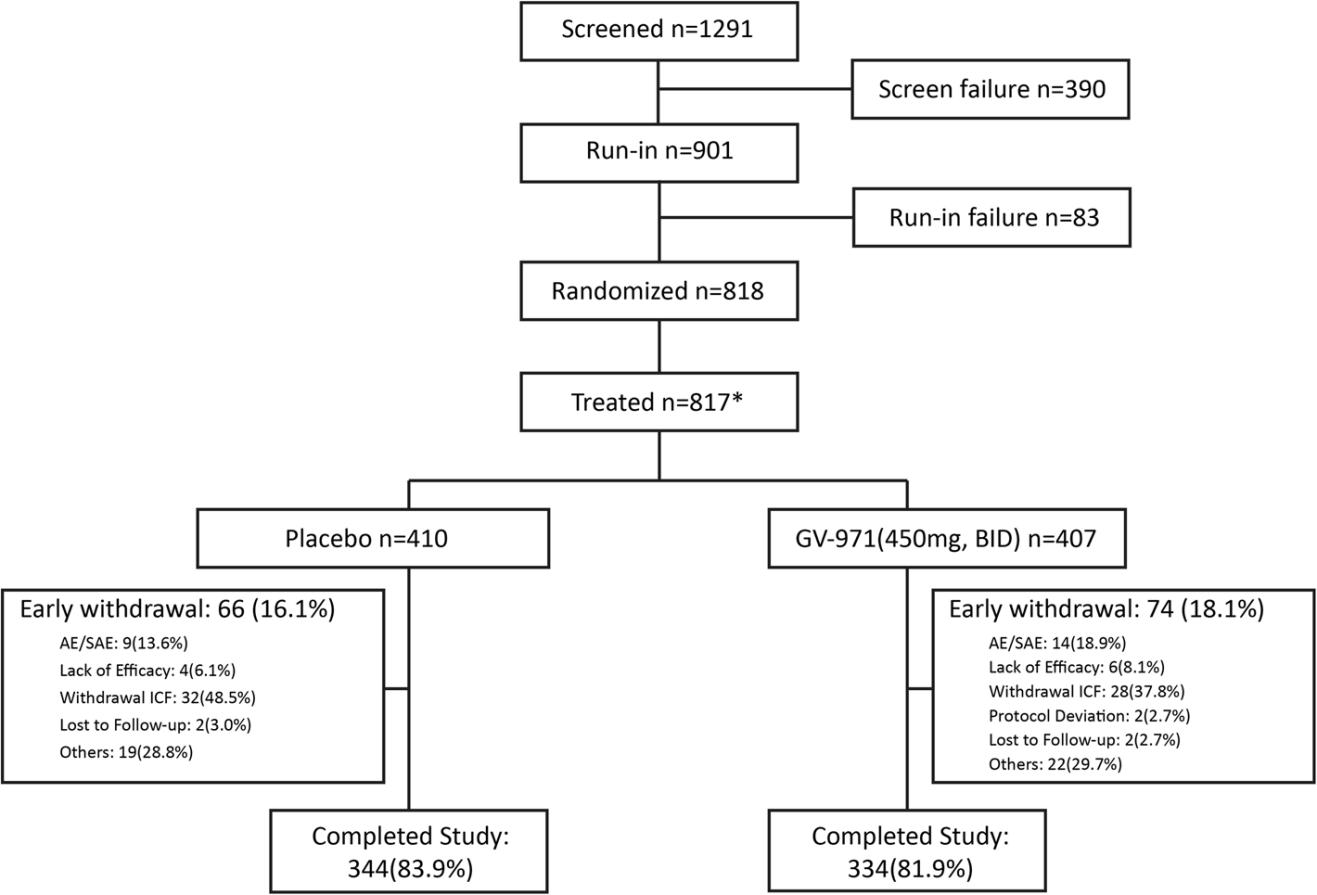
Enrollment, randomization, and completion. *Not treated *n* = 1, in GV-971 group

The screening, randomization, and follow-up of participants are summarized in Fig. 1. The trial was initiated in March 2014 and completed in June 2018. The trial was completed as planned after the last participant randomized had completed the exposure period and final visit measures. Among the 1291 participants screened, 818 participants were randomized, and 817 received at least one dose of the study treatment (408 were assigned randomly to the GV-971 group and 410 to the placebo group). Screen failures were 390 (30.2%). A total of 901 participants were included in the run-in period. Run-in failures were 83 (6.4%). The two main reasons for screen failure and run-in failure were that (1) MRI findings did not meet the inclusion criteria with 205 (43.3% of screen failures) and (2) 70 (14.8% of screen failures) participants/legal guardians withdrew consent (Fig. 1). The completion rate was 81.9% for participants in the GV-971 group and 83.9% for the placebo group. The most common reasons for early termination were withdrawal of consent and AEs, regardless of group assignment (Fig. 1).

### Baseline data

The demographic and baseline characteristics of the trial cohort are summarized in Table 1. There were no significant differences between the GV-971 and placebo groups with respect to age, sex, ethnicity, or education level (Table 1). In total, 52.5% of participants in the GV-971 group and 48.5% in the placebo group were apolipoprotein E epsilon 4 (APOE ε4) carriers. The mean ± SD MMSE score was 19.4 ± 4.4 in the GV-971 group and 19.5 ± 4.5 in the placebo group. The mean ± SD duration (in months) from symptom onset was 30.42 ± 20.59 in the GV-971 group and 31.46 ± 20.79 in the placebo group. There were no significant differences between the groups in baseline characteristics including age, race, education, APOE4 distribution, ADAS-Cog12, ADCS-ADL, and NPI scores.

**Table 1:** Demographic and baseline clinical characteristics

Characteristic	Placebo, *n* = 410 (%)	GV-971, *n* = 408 (%)	*p* value
Age—years	69.7 ± 8.20	69.6 ± 8.12	0.7374 [b]
≤ 65 years	131 (32.0%)	132 (32.4%)	0.9021 [c]
> 65 years	279 (68.0%)	276 (67.6%)	
Sex			0.8242 [c]
Male—no. (%)	177 (43.2%)	173 (42.4%)	
Female—no. (%)	233 (56.8%)	235 (57.6%)	
Han ethnicity—no. (%)	402 (98.0%)	398 (97.5%)	0.6261 [c]
Education—years			0.7394 [c]
> 6 years	335 (81.7%)	337 (82.6%)	
≤ 6 years	75 (18.3%)	71 (17.4%)	
APOE ε4 carrier—no. (%)	199 (48.5%)	214 (52.5%)	0.2758 [c]
MMSE score	19.5 ± 4.5	19.4 ± 4.4	0.5795 [b]
MMSE < 11	1 (0.2%)	0	0.6815 [d]
11 ≤ MMSE < 15	72 (17.6%)	68 (16.7%)	
15 ≤ MMSE ≤ 19	118 (28.8%)	122 (29.9%)	
20 ≤ MMSE ≤ 26	219 (53.4%)	216 (52.9%)	
26 < MMSE	0	2 (0.5%)	
Duration since symptom onset (months)	31.46 ± 20.79	30.42 ± 20.59	0.6087 [b]
ADAS-Cog12	20.88 ± 10.00	21.28 ± 10.14	0.5638 [b]
ADCS-ADL	64.2 ± 10.1	64.0 ± 11.2	0.8910 [b]
NPI	5.9 ± 8.6	5.6 ± 8.0	0.5651 [b]

[a] refers to the *t* test; [b] refers to the Wilcoxon rank sum test; [c] refers to the chi-square test; [d] refers to Fisher’s exact test

### Outcomes

**Fig. 2:**
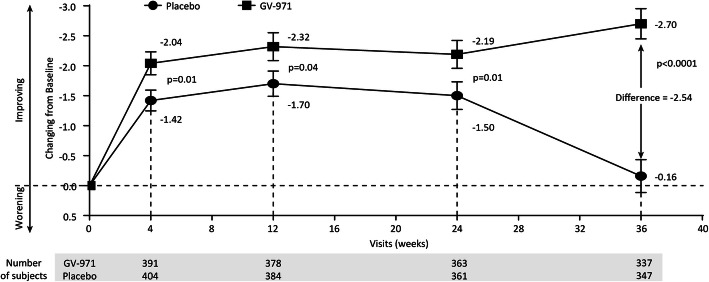
Mean ADAS-Cog12 score change from baseline at weeks 4, 12, 24, and 36 (observed value). The mean change from baseline to week 36 on the ADAS-Cog12 (with scores ranging from 0 to 75 and higher scores indicating greater impairment) by full analysis set was showed. Error bars represent standard errors (SE). *p* values are obtained from the Wilcoxon rank sum tests

**Fig. 3:**
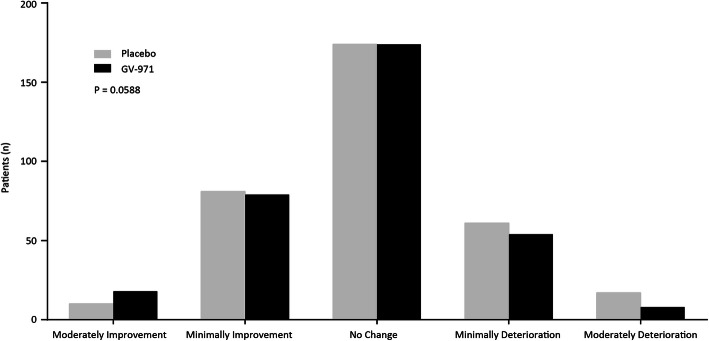
Secondary outcome of CIBIC+ at week 36. *p* value is obtained from a stratified Cochran-Mantel-Haenszel (CMH) test, including stratification factors of MMSE at baseline, education level, and age group

**Fig. 4:**
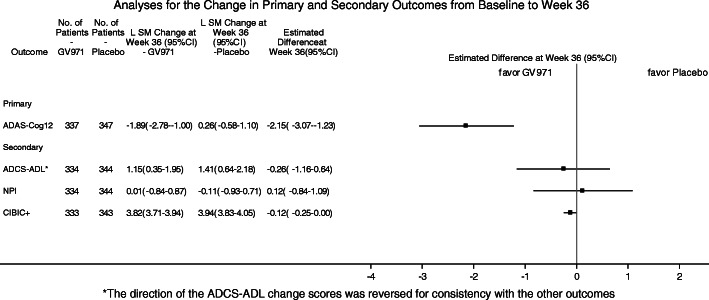
Analyses for the change in primary and secondary outcomes from baseline to week 36. *The direction of the ADCS-ADL change scores was reversed for consistency with the other outcomes

The results of the primary and secondary analyses are summarized in Table 2 and Figs. 2, 3, and 4. Changes from baseline over time in ADAS-cog12 scores are shown in Fig. 2. The mean changes from baseline at week 36 were − 2.70 points for the GV-971 group and − 0.16 points for the placebo group, with an unadjusted group difference (GV-971 group minus placebo group) of − 2.54 points. The mean modeled difference between the groups (GV-971 group minus placebo group) with regard to the change from baseline to week 36 was − 2.15 points (95% confidence interval, − 3.07 to − 1.23; *p* < 0.0001, with Cohen’s *d* effect size 0.53, using pooled SD of change score ANCOVA analysis. There were no significant drug-placebo differences for prespecified secondary outcomes. The *p* value of CIBIC+ was 0.059 between the groups (Figs. 3 and 4). The ADCS-ADL was directionally in favor of GV-971 but was not statistically different between the groups (LS difference = 0.26, *p* = 0.57) (Figs. 3 and 4). The NPI was directionally in favor of placebo but was not statistically different between the groups (LS difference = 0.12, *p* = 0.80) (Figs. 3 and 4).

**Table 2:** Primary and secondary outcomes

Outcome	Raw score at baseline		Raw score at week 36		Least square mean change at week 36 (95%CI)		Estimated difference at week 36 (95% CI)	*p* value
	Placebo	GV-971	Placebo	GV-971	Placebo	GV-971		
*Primary*								
ADAS-Cog12*	20.83 ± 10.03	21.30 ± 10.12	20.55 ± 11.93	18.32 ± 10.71	0.26 (− 0.58, 1.10)	−1.89 (− 2.78, − 1.00)	− 2.15 (3.07, − 1.23)	< 0.0001
*Secondary*								
CIBIC+**	–	–	4.0 ± 0.85	3.9 ± 0.83				0.0588
ADCS-ADL	64.2 ± 10.1	64.0 ± 11.2	63.4 ± 11.4	63.5 ± 11.6	−1.41 (−2.18, − 0.64)	−1.15 (− 1.95, − 0.35)	0.26 (−0.64, 1.16)	0.57
NPI	5.9 ± 8.7	5.6 ± 8.0	4.8 ± 8.6	5.0 ± 7.7	−0.11 (− 0.93, 0.71)	0.01 (− 0.84, 0.87)	0.12 (− 0.84, 1.09)	0.80

The plus-minus values for the scores at baseline and at 36 weeks are means (± SD). Least square means are estimated using ANCOVA, with the treatment group, education level, pooled center, and age group as fixed effects, and baseline MMSE level and baseline score as covariates. The estimated difference is the least square mean change from baseline between the two groups (GV-971 minus placebo group) at week 36. Differences may not calculate as expected because of rounding. CI denotes confidence interval

*Missing data handled using control-based pattern imputation

**Refer to Fig. 3. *p* value is obtained from a stratified Cochran-Mantel-Haenszel (CMH) test, including stratification factors of MMSE at baseline, education level, and age group

**Fig. 5:**
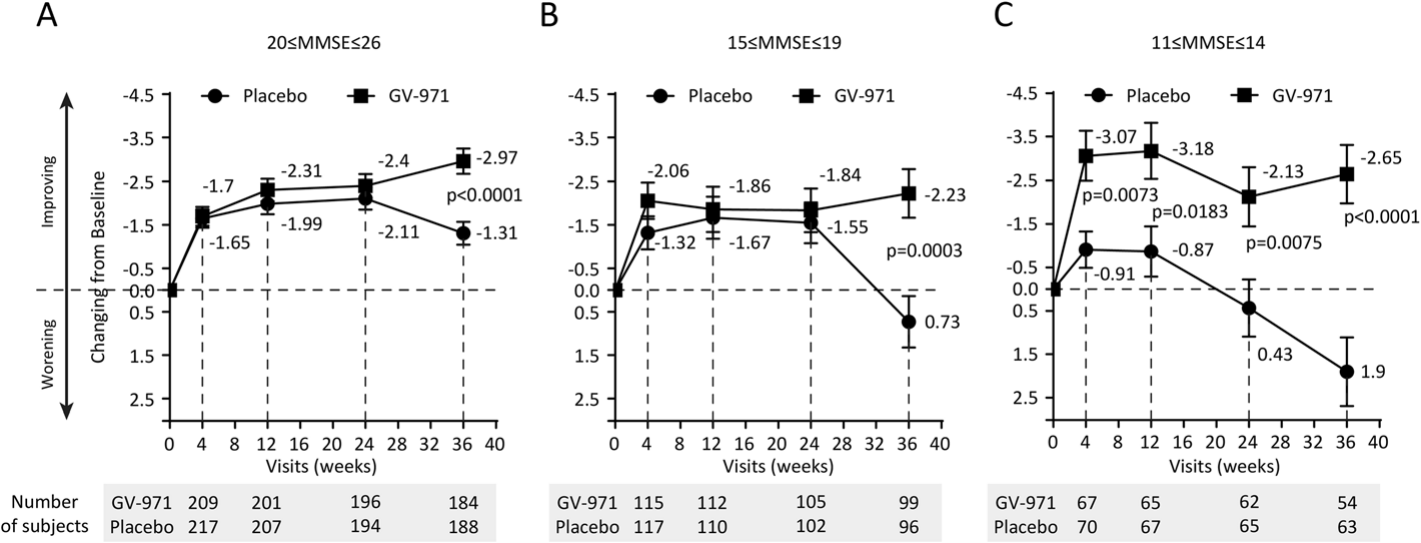
Mean ADAS-Cog12 score change from baseline at weeks 4, 12, 24, and 36 (observed value) on the ADAS-Cog12 by the MMSE subgroup. Error bars represent standard errors (SE). *p* values are obtained from the Wilcoxon rank sum tests

In pre-planned exploratory analyses, we assessed the treatment effect of GV-971 in the participant groups of MMSE 11–14, 15–19, and 20–26 (Fig. 5). The adjusted difference values of the primary outcome in ADAS-Cog12 between the groups were 4.55, 2.96, and 1.66, respectively (Fig. 5). Prespecified subgroup analyses for the change in ADAS-Cog12 score from baseline to week 36 are shown in Figure S1 (Supplementary Appendix). Significant intergroup differences in drug-placebo changes from baseline were found for all subgroups: ± APOE 4 allele, age (< 65; > 65 years) group, sex, education level (< 6; > 6 years), and MMSE score (3 terciles). In a post hoc subgroup analyses, significant intergroup differences were detected for CIBIC+ outcome in participants with MMSE scores 11–14 (*p* = 0.017), effect size 1.3. (Figure S2 in the Appendix).

Forty-one (10.5%) participants in the GV-971 group and 31 (7.7%) participants in the placebo group were assessed by [^18^F]-FDG-PET. The demographic and baseline characteristics were balanced between the placebo and GV-971 sub-cohort groups. There was no intergroup difference in the changes from baseline in the predefined sROI-based global relative CMRglu on [^18^F]-FDG-PET after 36 weeks of double-blind treatment.

### Adverse events

In total, 307 of 407 participants (75.4%) in the GV-971 group and 303 of 410 (73.9%) in the placebo group had at least one treatment-emergent adverse event (TEAE) in the safety analysis set. Table 3 lists the TEAEs that occurred in ≥5% of participants. Among the common TEAEs, hyperlipidemia and nasopharyngitis were higher in the GV-971 group than in the placebo group. All other common TEAEs were not statistically significantly different between the two groups.

**Table 3:** TEAEs that occurred in 5% or more of the subjects in the study

System organ class	Placebo, *n* = 410		GV-971 900 mg, *n* = 407	
	Number of events	*n* (%)	Number of events	*n* (%)
Infectious and infectious diseases				
Urinary tract infection	45	40 (9.8%)	31	30 (7.4%)
Nasopharyngitis	26	23 (5.6%)	34	30 (7.4%)
Upper respiratory tract infection	36	30 (7.3%)	29	23 (5.7%)
Various types of inspection				
Elevated blood glucose	30	29 (7.1%)	32	28 (6.9%)
Various nervous system diseases				
Dizzy	28	24 (5.9%)	26	23 (5.7%)
Metabolic and nutritional diseases				
Hyperlipidemia	15	14 (3.4%)	29	29 (7.1%)

Seventy-six participants (18.7%) in the GV-971 group and 86 (20.9%) in the placebo group reported a TEAE that was related or possibly related to the trial drug according to an investigator. Fourteen participants (3.4%) in the GV-971 group and 9 (2.2%) in the placebo group had a TEAE that led to their discontinuation from the trial.

Thirty-three participants (8.1%) in the GV-971 group and 29 (7.1%) in the placebo group reported at least one serious adverse event (SAE). For the GV-971 group, the SAE of infectious pneumonia reported by one participant was determined as being possibly related to the trial drug by investigators. The remaining SAEs were determined to be not related or possibly related to the trial drug. Tables S1-1 and S1-2 in the Supplementary Appendix list all the SAEs that occurred for each treatment group.

Two participants in the GV-971 group died during the trial because of metastatic lung cancer and brain stem encephalitis (Table S2 in the Supplementary Appendix). Examination of all listed causes of death revealed no similarities, and the deaths were not reported to be related to the drug as assessed by the site investigator.

## Discussion

We report the first phase 3 clinical trial of GV-971 that demonstrated a robust, statistically significant drug-placebo difference in change from baseline favoring GV-971 as measured by the ADAS-cog. The drug-placebo difference was present at the first assessment and continued throughout the 36-week trial. The separation was greatest at the endpoint of the trial between groups. In this trial, GV-971 had a therapeutic effect on patients with mild-to-moderate AD. The effects were most pronounced in those with more severe cognitive decline. The trial demonstrated improvement above baseline suggesting that GV-971 has symptomatic effects, while the non-clinical studies support the occurrence of disease-modifying effects in concert with the symptomatic changes.

The trial was conducted according to the International Conference on Harmonization (ICH) of Drug Development Standards and Good Clinical Practice (GCP) guidelines, was organized and monitored by an international contract research organization (CRO), required centralized reading of MRI to confirm temporal lobe atrophy consistent with AD, and included extensive training in clinical trial methods by international vendors. GV-971 is an oligosaccharide with a novel proposed mechanism of action. In non-clinical studies, GV-971 showed a reduction of neuroinflammation in the brain by regulating the gut microbiota and reducing peripheral inflammation that may aggravate neuroinflammation. GV-971 directly binds to Aβ and decreases Aβ deposition in the brain [11–15]. The effect of GV-971 on the gut mirobiota and neuroinflammation is rapid, occurring within 4 weeks in animal models, which might contribute to the early therapeutic effect of GV-971 observed on the ADAS-Cog12. The effect on Aβ deposition requires a longer exposure but could work synergistically with the effect on the gut mirobiota and neuroinflammation. Emerging evidence supports the concept that dysbiosis of the gut microbiota, neuroinflammation, Aβ deposition, and tau hyperphosphorylation can interact to accelerate AD progression [32–35]. The therapeutic effect of GV-971 on dysbiosis of the gut microbiota, neuroinflammation, and Aβ deposition may ameliorate the pathologic cascade to improve symptoms and delay disease progression with beneficial longer-term effects. The rapid decline in the placebo group at the end of the trial and the sustained response to GV-971 during the course of the trials contribute to the growing drug-placebo difference as the trial progressed. Investigation of the effects of GV-971 will require additional trials and inclusion of biomarkers to verify the mechanism of action.

In the full analysis set, none of the secondary endpoints, i.e., global function (CIBIC+), activities of daily living (ADCS-ADL) scale or behavioral symptoms (NPI), showed significant drug-placebo differences. The lack of significance on the CIBIC+, ADCS-ADL, and NPI might be attributable to the limited sample size, which was calculated based on the primary endpoint. Cultural differences may also have contributed to these negative outcomes as activities of daily living, and behavioral assessments are subject to many cultural interpretations [25]. NPI scores at baseline were very low (average score, 3 points) leaving a limited dynamic range to show measurable improvement. Finally, the relatively short duration of the trial may have contributed to the inability to show a change from baseline or drug-placebo difference.

### Limitations

A limitation of our trial was the lack of requirement for the presence of a diagnostic amyloid biomarker at screening, thereby potentially allowing participants who had dementia owing to non-amyloid-related diseases to be included. Amyloid positron emission tomography was not widely available in China at the time the trial was planned and initiated. Approximately 50% of the participants in the trial were carriers of the APOE4 gene and therefore had a higher likelihood of amyloid-related disease than non-carriers [5, 36]. Treatment benefit was evident in both APOE4 carriers and non-carriers. Structural MRI verifying the presence of temporal lobe atrophy based on central reads of the imaging supported the diagnosis of AD. Other limitations include that the trial was conducted in one country and the external validity of the findings remains to be demonstrated. The unusually robust placebo responses observed in this trial may have been related to “trial effects,” including the higher proportion of mild cases, and the excellent care delivery in trials and high participant and caregiver expectations. Similar placebo effects and benefits have been observed in other trials in China [37–40].

## Conclusions

In conclusion, this phase 3 trial was well conducted by the International Conference on Harmonization of Drug Development and Good Clinical Practice Guidelines. The trial demonstrated a robust and sustained drug-placebo difference on the primary outcome. We plan to conduct a global phase 3 trial to assess the safety and efficacy of GV-971 in additional populations. We will collect biomarkers in these trials and will interrogate the mechanism of action of GV-971 including its effects on neuroinflammation and gut dysbiosis.

## Supplementary Information

**Additional file 1:** Supplementary figures and tables.

## Data Availability

The datasets used and/or analyzed during the present study are available from the corresponding author on reasonable request.

## Abbreviations

AD: Alzheimer’s disease
ADAS-cog12: 12-item cognitive subscale of the Alzheimer’s Disease Assessment Scale
CIBIC+: Clinician’s Interview-Based Impression of Change with caregiver input
ADCS-ADL: Alzheimer’s Disease Cooperative Study-Activities of Daily Living
NPI: Neuropsychiatric Inventory
FDG-PET: Fluorodeoxyglucose-positron emission tomography
MRI: Magnetic resonance imaging
MMSE: Mini-Mental State Examination
APOE: Apolipoprotein E (APOE)
ALT: Alanine transaminase
AST: Aspartate transaminase
NMPA: National Medical Products Administration
GMP: Good Manufacturing Practice
IWRS: Interactive web response system
CMRglu: Cerebral metabolic rate for glucose
AEs: Adverse events
ANCOVA: Analysis of covariance
MMRM: Mixed-model repeated-measures model
FAS: Full analysis set
SUVR: Standardized uptake value ratio
APOE ε4: Apolipoprotein E epsilon 4
TEAE: Treatment-emergent adverse event
SAE: Serious adverse event
ICH: International Conference on Harmonization
GCP: Good Clinical Practice
CRO: Contract research organization
Aβ: β-Aamyloid
