# Supplementary material for: A 36-week multicenter, randomized, double-blind, placebo-controlled, parallel-group, phase 3 clinical trial of sodium oligomannate for mild-to-moderate Alzheimer’s dementia
Source: Alzheimers Res Ther. 2021 Mar 17;13:62. doi: 10.1186/s13195-021-00795-7 (PMC7967962; doi:10.1186/s13195-021-00795-7)
Supplement: Supplementary file 1 — Additional file 1: Supplementary figures and tables. [file 13195_2021_795_MOESM1_ESM.docx]

**SUPPLEMENTARY APPENDIX**

Supplemental materials for

A 36-week Multicenter, Randomized, Double-blind, Placebo-controlled, Parallel-group, Phase 3 Clinical Trial of Sodium Oligomannate for Mild-to-Moderate Alzheimer’s Dementia

**Table of contents**

| **Contents** | **Page** |
| --- | --- |
| List of investigators | 2-3 |
| Figure S1 Subgroup Analyses for the Change in ADAS-Cog12 Score from Baseline to Week 36 | 4 |
| Figure S2 Subgroup Analyses of Secondary Outcomes | 5 |
| Table S1 Serious Adverse Events - GV-971 Subjects | 5-9 |
| Table S2 Deaths | 10 |
| Figure S3 Study Flowchart | 11 |
| CONSORT 2010 Flow Diagram | 12 |

**List of investigators**

Shifu Xiao, M.D., Ph.D.^1#*^, Piu Chan, M.D., Ph.D.^2#^, Tao Wang, M.D., Ph.D.^1#^, Zhen Hong, M.D.^3^, Shuzhen Wang, M.D.^4^, Weihong Kuang, M.D.^5^, Jincai He, M.D.^6^, Xiaoping Pan, M.D.^7^, Yuying Zhou, M.D.^8^, Yong Ji, M.D., Ph.D.^8^, Luning Wang, M.D., Ph.D.^9^, Yan Cheng, M.D., Ph.D.^10^, Ying Peng, M.D.^11^, Qinyong Ye, Ph.D.^12^, Xiaoping Wang, M.D., Ph.D.^13^, Yuncheng Wu, M.D., Ph.D.^13^, Qiumin Qu, M.D.^14^, Shengdi Chen, M.D., Ph.D.^15^, Shuhua Li, M.D.^16^, Wei Chen, M.D.^17^, Jun Xu, M.D., Ph.D.^18^, Dantao Peng, M.D., Ph.D.^19^, Zhongxin Zhao, M.D.^20^, Yansheng Li, M.D.^21^, Junjian Zhang, M.D.^22^, Yifeng Du, Ph.D.^23^, Weixian Chen, Ph.D.^24^, Dongsheng Fan, M.D.^25^, Yong Yan, M.D.^26^, Xiaowei Liu, M.D.^27^, Wei Zhang, M.D., Ph.D.^28^, Benyan Luo, M.D., Ph.D.^29^, Wenyuan Wu, Ph.D.^30^, Lu Shen, M.D., Ph.D.^31^, Chunfeng Liu, M.D.^32^, Peixian Mao, M.D.^33^, Qiumei Wang, M.D.^34^, Qianhua Zhao, M.D., Ph.D.^3^, Qihao Guo, M.D., Ph.D.^3^, Yongtao Zhou, M.D., Ph.D.^2^, Yi Li, M.D., Ph.D.^4^, Lijun Jiang, M.D.^5^, Wenwei Ren, M.D.^6^, Yingjun Ouyang, M.D.^7^, Yan Wang, M.D.^8^, Shuai Liu, M.D.^8^, Jianjun Jia, M.D., Ph.D.^9^, Nan Zhang, M.D.^10^, Zhonglin Liu, M.D.^11^, Raoli He, M.D.^12^, Tingyi Feng, M.D.^13^, Wenhui Lu, M.D.^14^, Huidong Tang, M.D., Ph.D.^15^, Ping Gao, M.D.^16^, Yingchun Zhang, M.D.^17^, Lanlan Chen, M.D.^18^, Lei Wang, M.D.^19^, You Yin, M.D., Ph.D.^20^, Qun Xu, M.D., Ph.D.^21^, Jinsong Xiao, M.D.^22^, Lin Cong, Ph.D.^23^, Xi Cheng, M.D.^24^, Hui Zhang, M.D.^25^, Dan Gao, M.D.^26^, Minghua Xia, M.D.^27^, Tenghong Lian, M.D., Ph.D.^28^, Guoping Peng, M.D., Ph.D.^29^, Xu Zhang, M.D.^30^, Bin Jiao, M.D., Ph.D.^31^, Hua Hu, Ph.D.^32^, Xueyan Chen, M.D.^33^, Yihui Guan, M.D.^3^, Ruixue Cui, M.D. ^34^, Qiu Huang Ph.D. ^35^, Xianliang Xin, Ph.D. ^36^, Hongjian Chen ^36^, Yu Ding ^36^, Jing Zhang Ph.D. ^36^, Teng Feng Ph.D. ^36^, Marc Cantillon M.D. ^36^, Kewei Chen Ph.D. ^37^, Jeffrey L. Cummings M.D. , Sc.D ^38^, Jian Ding, Ph.D. ^39^, Meiyu Geng, Ph.D.^39*^, Zhenxin Zhang, M.D. ^34*^

^#^Contributed equally.

^1^Department of Geriatric Psychiatry, Shanghai Mental Health Centre, Shanghai Jiaotong University School of Medicine, Shanghai, China; Alzheimer’s Disease and Related Disorders Center, Shanghai Jiao Tong University, Shanghai, China; ^2^Xuanwu Hospital Capital Medical University, Beijing, China; ^3^Huashan Hospital, Fudan University, Shanghai, China; ^4^Qilu Hospital of Shandong University, Ji’nan, China; ^5^West China Hospital of Sichuan University, Chengdu, China; ^6^The First Affiliated Hospital of Wenzhou Medical University, Wenzhou, China; ^7^Guangzhou First People's Hospital, School of Medicine, South China University of Technology, Guangzhou, China; ^8^Tianjin Huanhu Hospital; Huanhu Hospital Affiliated to Nankai University, Tianjin, China; ^9^Department of Geriatric Neurology of PLA General Hospital,Beijing, China; ^10^Department of Neurology, Tianjin Medical University General Hospital, Tianjin, China; ^11^Sun Yat-Sen Memorial Hospital, Sun Yat-Sen University, Guangzhou, China; ^12^Fujian Medical University Union Hospital, Fuzhou, China; ^13^Department of Neurology, Shanghai General Hospital, Shanghai Jiao Tong University School of Medicine, Shanghai, China; ^14^Department of Neurology, The First Affiliated Hospital of Xi’an Jiaotong University，Xi'an, China; ^15^Department of Neurology, Ruijin Hospital, Shanghai Jiaotong University School of Medicine, Shanghai, China; ^16^Beijing Hospital, Beijing, China; ^17^Department of Psychiatry, Sir Run Run Shaw Hospital, Zhejiang University School of Medicine, and Key Laboratory of Medical Neurobiology of Zhejiang Province, Hangzhou, China; ^18^Northern Jiangsu People's Hospital, Yangzhou, China; ^19^Department of Neurology, China-Japan Friendship Hospital, Beijing, China; ^20^Shanghai Changzheng Hospital, Shanghai, China; ^21^Renji Hospital, Shanghai Jiaotong University School of Medicine, Shanghai, China; ^22^Department of Neurology, Zhongnan Hospital of Wuhan University, Wuhan, China; ^23^Shandong Provinical Hospital affiliated to Shandong University, Ji’nan, China; ^24^Jiangsu Province People's Hospital, Nanjing, China; ^25^Peking University Third Hospital, Beijing, China; ^26^The First Affiliated Hospital of Chongqing Medical University, Chongqing, China; ^27^Department of Geriatric psychiatry, Wuxi Mental Health Center, Wuxi, China; ^28^Department of Neurology, Beijing Tiantan Hospital, Capital Medical University, Beijing, China; ^29^The First affiliated hospital, Zhejiang University School of Medicine, Hangzhou, China; ^30^Tongji Hospital of Tongji University, Shanghai, China; ^31^Xiangya Hospital Central South University, Changsha, China; ^32^The Second Affiliated Hospital of Soochow University, Suzhou, China; ^33^Beijing An Ding Hospital, Capital Medical University, Beijing, China; ^34^Peking Union Medical College Hospital, Beijing, China; ^35^Med-X Research Institution, Shanghai Jiao Tong University, Shanghai, China; ^36^Shanghai Green Valley Pharmaceutical Co. Ltd., No. 421, Niudun Road, Shanghai, China; ^37^Banner Alzheimer’s Institute, Phoenix, AZ, USA. ^38^Departmen of Brain Health, School of Integrated Health Sciences, University of Nevada Las Vegas; ^39^State Key Laboratory of Drug Research, Shanghai Institute of Materia Medica, Chinese Academy of Sciences, Shanghai, China

**^*^Corresponding Author:**

Shifu Xiao, M.D., Ph.D., Prof. and Dir., Department of Geriatric Psychiatry, Shanghai Mental Health Centre, Shanghai Jiaotong University School of Medicine, 600 South Wan Ping Road, Shanghai 200030, China. Alzheimer’s Disease and Related Disorders Center, Shanghai Jiao Tong University, Shanghai, China.

**EMAIL:** [**xiaoshifu@msn.com**](mailto:xiaoshifu@msn.com)

MeiyuGeng, Ph.D., Professor of State Key Laboratory of Drug Research, Shanghai Institute of Materia Medica, Chinese Academy of Sciences, 555 Zuchongzhi Road, Shanghai, China. TEL: (86)-21-50806600

**EMAIL: mygeng@simm.ac.cn**

Zhenxing Zhang, MD., Peking Union Medical College Hospital, No.1 Shuaifuyuan, Beijing 100730,China. Tel: (86)-10-69155095

**EMAIL: wuzhangzhenxin@163.com**

# SUPPLEMENTAL TABLES AND FIGURES

| **Figure S1 Subgroup Analyses for the Change in ADAS-Cog12 Score from Baseline to Week36** |
| --- |

CI denotes confidence interval; LSM denotes least squares means; SD denotes standard deviation; SEM denotes standard error of mean In the subgroup analyses, significant intergroup differences were detected of CIBIC+ in the subjects with the lowest MMSEs (MMSE 11-14) (p=0.0174).

| **Figure S2 Subgroup Analyses of Secondary Outcomes** |
| --- |

A total of 33 subjects (8.1%) in the GV-971 group reported at least one serious adverse event.

| **Table S1-1 Serious Adverse Events - GV-971 Subjects** | | | | | | | | |
| --- | --- | --- | --- | --- | --- | --- | --- | --- |
| **No.** | **Screening #** | **SAE Term** | **Start Date** | **End Date** | **Severity** | **Relationship with IP** | **Action on IP** | **Withdrawal due to SAE** |
| 1 | 122080 | pneumonia | 2017-11-26 | 2018-01-05 | Moderate | Possibly related | No change | N |
| 2 | 101033 | pneumonia | 2016-04-UNK | 2016-05-06 | Mild | possibly unrelated | No change | N |
| 3 | 101057 | cerebral infarction | 2016-09-05 | 2016-09-12 | Moderate | possibly unrelated | IP discontinuation | Y |
| 4 | 101093 | gallstone with chronic cholecystitis | 2017-09-29 | 2018-01-13 | Severe | possibly unrelated | NA | N |
| 5 | 101100 | biliary pancreatitis | 2017-10-30 | 2017-11-09 | Severe | possibly unrelated | IP interruption | N |
| 6 | 101100 | pectal sigmoid colon ulcer with perforation | 2017-12-28 | 2018-01-11 | Moderate | possibly unrelated | IP interruption | N |
| 7 | 102059 | anti-GQ1b antibody syndrome | 2017-01-12 | 2017-01-20 | Moderate | possibly unrelated | IP discontinuation | Y |
| 8 | 104005 | gastrointestinal schwannoma | 2015-02-27 | 2015-06-19 | Moderate | possibly unrelated | IP discontinuation | Y |
| 9 | 115016 | facial cellulitis | 2016-05-07 | 2016-05-17 | Severe | possibly unrelated | No change | N |
| 10 | 121014 | lumbar fracture | 2015-11-01 | 2015-12-09 | Moderate | possibly unrelated | No change | N |
| 11 | 123012 | left leg fracture | 2016-02-02 | 2016-02-12 | Severe | possibly unrelated | IP interruption | Y |
| 12 | 124011 | cataract aggravation | 2016-05-18 | 2016-05-25 | Severe | possibly unrelated | No change | N |
| 13 | 126006 | cholecystitis | 2016-07-08 | 2016-07-20 | Moderate | possibly unrelated | No change | N |
| 14 | 128015 | constipation | 2017-07-20 | 2017-07-27 | Mild | possibly unrelated | IP discontinuation | Y |
| 15 | 129015 | colon cancer | 2017-01-03 | 2017-01-13 | Severe | possibly unrelated | NA | N |
| 16 | 135009 | unstable angina | 2016-07-13 | 2016-07-21 | Moderate | possibly unrelated | No change | N |
| 17 | 101072 | proximal right humerus fracture | 2016-06-30 | 2016-07-18 | Moderate | unrelated | NA | N |
| 18 | 101072 | atrial fibrillation | 2016-09-08 | 2016-09-23 | Moderate | unrelated | IP discontinuation | Y |
| 19 | 102004 | lumbar disc herniation | 2014-11-20 | 2014-12-18 | Moderate | unrelated | No change | N |
| 20 | 102045 | colon polyps | 2016-02-29 | 2016-03-04 | Moderate | unrelated | No change | N |
| 21 | 105004 | metastatic lung cancer | 2015-04-12 | 2015-08-04 | Severe | unrelated | IP discontinuation | Y |
| 22 | 109013 | aggravated congenital heart disease- aortic stenosis | 2015-12-15 | 2016-01-11 | Moderate | unrelated | IP interruption | N |
| 23 | 109019 | duodenal bulb ulcer (S2 phase) | 2015-10-27 | 2015-11-09 | Severe | unrelated | NA | N |
| 24 | 115021 | gastric cancer | 2016-09-26 |  | Severe | unrelated | IP discontinuation | Y |
| 25 | 116027 | benign paroxysmal positional vertigo | 2018-01-03 | 2018-01-08 | Mild | unrelated | No change | N |
| 26 | 117014 | brain stem encephalitis | 2016-03-16 | 2016-04-11 | Severe | unrelated | IP discontinuation | Y |
| 27 | 117045 | upper respiratory tract infection | 2017-09-20 | 2017-09-29 | Mild | unrelated | No change | N |
| 28 | 118005 | rectal adenoma | 2014-11-18 | 2015-01-08 | Mild | unrelated | IP interruption | N |
| 29 | 121028 | acute coronary syndrome | 2017-04-13 | 2017-04-19 | Moderate | unrelated | IP discontinuation | Y |
| 30 | 122053 | cerebral infarction | 2016-03-07 | 2016-03-20 | Moderate | unrelated | IP interruption | N |
| 31 | 122091 | diabetic | 2017-05-28 | 2017-06-11 | Mild | unrelated | No change | N |
| 32 | 124008 | right lower extremity venous insufficiency | 2016-06-06 | 2016-06-24 | Mild | unrelated | No change | N |
| 33 | 125015 | posterior circulation ischemia | 2016-02-07 | 2016-02-23 | Mild | unrelated | No change | N |
| 34 | 126012 | acute asthma attack | 2016-04-05 | 2016-04-14 | Moderate | unrelated | No change | N |
| 35 | 126012 | bronchiectasis | 2016-10-11 | 2016-10-25 | Moderate | unrelated | No change | N |
| 36 | 126012 | bronchial infection | 2016-10-11 | 2016-10-25 | Moderate | unrelated | No change | N |
| 37 | 132033 | senile cataract in both eyes | 2016-05-23 | 2016-05-25 | Moderate | unrelated | No change | N |
| 38 | 132033 | left eye senile cataract | 2016-06-12 | 2016-06-13 | Moderate | unrelated | NA | N |
|  | | | | | | | | |

A total of 29 (7.1%) in the placebo group reported at least one serious adverse event.

| **Table S1-2 Serious Adverse Events – Placebo Subjects** | | | | | | | | |
| --- | --- | --- | --- | --- | --- | --- | --- | --- |
| **No.** | **Screening #** | **SAE Term** | **Start Date** | **End Date** | **Severity** | **Relationship with IP** | **Action on IP** | **Withdrawal due to SAE** |
| 1 | 125002 | intracerebral hematoma | 2015-02-04 | 2015-02-27 | Moderate | Possibly related | IP discontinuation | Y |
| 2 | 136005 | rash | 2017-09-01 | 2017-09-23 | Mild | Possibly related | IP interruption | Y |
| 3 | 102073 | right eye cataract | 2017-09-21 | 2017-10-31 | Mild | possibly unrelated | No change | N |
| 4 | 115026 | urinary tract infection | 2016-12-29 | 2017-01-02 | Moderate | possibly unrelated | No change | N |
| 5 | 115026 | left leg varicose veins | 2017-01-02 | 2017-01-11 | Mild | possibly unrelated | IP interruption | N |
| 6 | 115047 | erosive gastritis | 2017-06-14 | 2017-06-20 | Moderate | possibly unrelated | IP discontinuation | Y |
| 7 | 116015 | acute bronchitis | 2016-03-29 | 2016-04-04 | Moderate | possibly unrelated | No change | N |
| 8 | 118058 | hyponatremia | 2016-08-01 | 2016-08-02 | Severe | possibly unrelated | No change | N |
| 9 | 121024 | T 12, L3, 4, 5 compression fracture | 2016-06-07 | 2016-07-06 | Moderate | possibly unrelated | No change | N |
| 10 | 123024 | lower extremity arteriosclerosis obliterans | 2016-03-10 | 2016-03-16 | Moderate | possibly unrelated | No change | N |
| 11 | 123055 | left inguinal hernia | 2017-09-01 | 2017-09-05 | Moderate | possibly unrelated | NA | N |
| 12 | 124002 | Right femoral neck fracture | 2015-04-22 | 2015-06-28 | Severe | possibly unrelated | No change | N |
| 13 | 129011 | lacunar cerebral infarction | 2015-12-24 | 2016-01-21 | Moderate | possibly unrelated | IP discontinuation | Y |
| 14 | 131004 | diabetes with elevated blood sugar | 2016-02-29 | 2016-03-05 | Mild | possibly unrelated | No change | N |
| 15 | 134014 | hyperthyroidism | 2015-12-25 | 2016-01-14 | Severe | possibly unrelated | IP discontinuation | N |
| 16 | 134014 | left frontal cerebral infarction | 2015-12-25 | 2016-01-14 | Severe | possibly unrelated | IP discontinuation | N |
| 17 | 134046 | thoracic fracture | 2017-04-25 | 2017-04-30 | Moderate | possibly unrelated | No change | N |
| 18 | 135022 | rectal polyp | 2017-03-24 | 2017-03-28 | Moderate | possibly unrelated | No change | N |
| 19 | 105009 | cerebral infarction | 2015-10-30 | 2015-11-15 | Moderate | unrelated | IP discontinuation | Y |
| 20 | 107008 | brain stem infarction | 2015-10-09 |  | Severe | unrelated | IP discontinuation | Y |
| 21 | 108039 | hemangioma | 2016-05-18 | 2016-06-08 | Mild | unrelated | No change | N |
| 22 | 109005 | fracture | 2015-08-12 |  | Severe | unrelated | IP discontinuation | N |
| 23 | 109010 | lung infection | 2015-06-08 | 2015-06-18 | Moderate | unrelated | IP interruption | N |
| 24 | 113015 | left upper lung tongue adenocarcinoma | 2016-06-16 | 2016-06-26 | Moderate | unrelated | No change | N |
| 25 | 113015 | left lower lung sclerosinghemangioma | 2016-06-16 | 2016-06-26 | Moderate | unrelated | No change | N |
| 26 | 113016 | left femoral neck fracture | 2016-03-06 | 2016-05-18 | Moderate | unrelated | No change | Y |
| 27 | 113016 | right femoral neck fracture | 2016-11-03 | 2017-01-09 | Moderate | unrelated | NA | N |
| 28 | 115047 | anxiety disorder | 2017-07-14 | 2017-07-24 | Moderate | unrelated | No change | N |
| 29 | 117050 | scalp hematoma | 2017-11-10 | 2017-11-14 | Mild | unrelated | No change | N |
| 30 | 117052 | bronchial Asthma | 2017-10-02 | 2017-10-13 | Mild | unrelated | No change | N |
| 31 | 120004 | multiple fracture | 2015-04-10 | 2015-09-19 | Moderate | unrelated | IP interruption | N |
| 32 | 132005 | dengue fever | 2014-09-25 | 2014-09-27 | Mild | unrelated | IP interruption | N |
| 33 | 132025 | vertebral basilar artery insufficiency | 2015-12-07 | 2015-12-15 | Mild | unrelated | No change | N |
| 34 | 132043 | L-1 compression fracture | 2016-09-11 | 2016-09-23 | Moderate | unrelated | No change | N |
|  | | | | | | | | |

Three subjects in the GV-971 group died during the study; two died from TEAEs and 1 died during the follow-up period. Examination of all listed causes of death revealed no clear treatment-related pattern.

| **Table S2 Deaths** | | | | | | | | | | | |  |
| --- | --- | --- | --- | --- | --- | --- | --- | --- | --- | --- | --- | --- |
| **Group** | **Screening # #** | **Age** | **Gender** | **SAE Term** | **Start Date** | **End Date** | **Duration (day)** | **Relation-ship with IP** | **Severity** | **Action on IP** | **Randomized Date** | **Study days** |
| GV-971 | 105004 | 57 | M | metastatic lung cancer | 2015-04-12 | 2015-08-04 | 115 | unrelated | Severe | IP discontinu-ation | 2014/12/22 | 111 |
| GV-971 | 117014 | 67 | M | brain stem encephalitis | 2016-03-16 | 2016-04-11 | 27 | unrelated | Severe | IP discontinu-ation | 2015/9/9 | 189 |

**
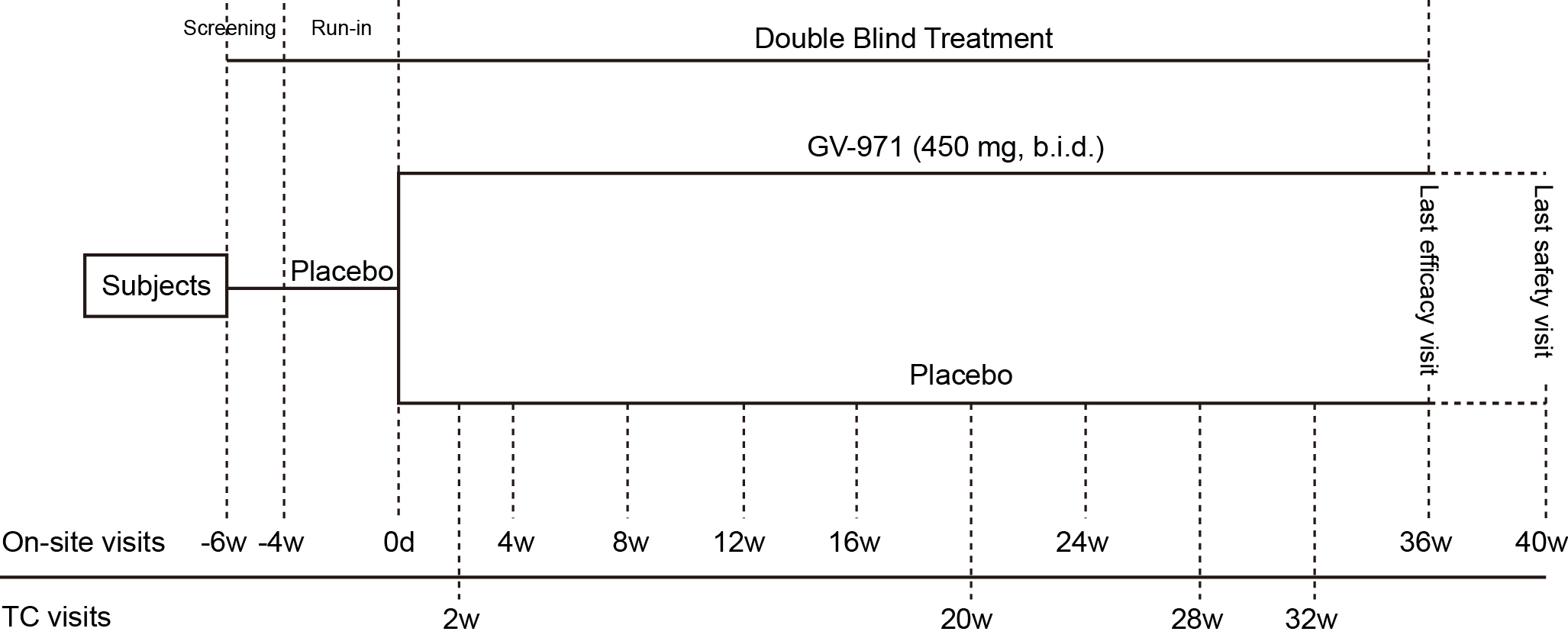
**

**Figure S3 Study Flowchart**

There were 10 on-site visits and 4 TC visits. In general, the run-in period was no less than 4 weeks. However, for naïve patients and those receiving no anti-AD drugs 3 months before screening, the run-in period could be shortened to 2 weeks. Interim analysis of safety was scheduled when all enrolled patients finished the visit at week 24.At week 24, MMSE was to be re-evaluated in case of obvious disease progression to determine the withdrawal of subjects from study.


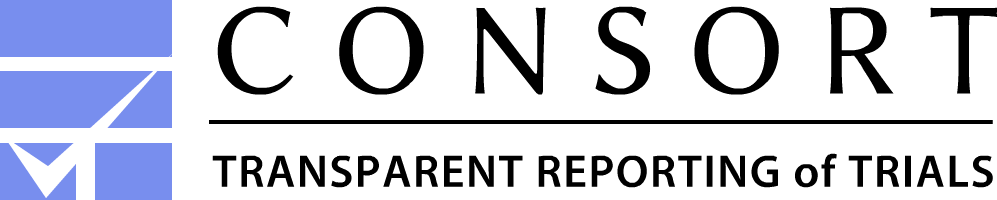


**CONSORT 2010 Flow Diagram**

Analysed (n=309)
Excluded from analysis (n=101)

Discontinued intervention (n=66)

Prohibited Medication (n=24)

Compliance not in 80%-120% (n=35)

Actual received drug KIT different from planned KIT (n=3)

Others (n=4)

Analysed (n=302)
Excluded from analysis (n=106)

Discontinued intervention (n=74)

Prohibited Medication (n=22)

Compliance not in 80%-120% (n=41)

Actual received drug KIT different from planned KIT (n=3)

Others (n=6)

Excluded (n=473)

♦  Not meeting inclusion criteria or met exclusion criteria (n=344)

♦  Declined to participate (n=51)

♦  Other reasons (n=78)

## Analysis

Lost to follow-up (give reasons) (n=2)

Discontinued intervention (n=64)

AE/SAE: 9

Lack of Efficacy: 4

Withdrawal ICF: 32

Lost to Follow-up: 2

Others: 19

Lost to follow-up (give reasons) (n=2)

Discontinued intervention (n=72)

AE/SAE: 14

Lack of Efficacy: 6

Withdrawal ICF: 28

Protocol Deviation: 2

Lost to Follow-up: 2

Others: 22

## Follow-Up

## Enrollment

Allocated to placebo (n=410)

♦ Received allocated intervention (n=410)

♦ Did not receive allocated intervention (n=0)

## Allocation

Allocated to GV-971 (n=408)

♦ Received allocated intervention (n=407)

♦ Did not receive allocated intervention (n=1)

Randomized (n=818)

Assessed for eligibility (n=1291)
